# Evolutionary development of the *Homo antecessor* scapulae (Gran Dolina site, Atapuerca) suggests a modern-like development for Lower Pleistocene *Homo*

**DOI:** 10.1038/s41598-021-83039-w

**Published:** 2021-02-18

**Authors:** Daniel García-Martínez, David J. Green, José María Bermúdez de Castro

**Affiliations:** 1 Centro Nacional para el Estudio de la Evolución Humana (CENIEH), Paseo Sierra de Atapuerca 3, 09002 Burgos, Spain; 2 Departamento de Paleobiología, Museo Nacional de Ciencias Naturales (CSIC), José Gutiérrez Abascal 2, 28006 Madrid, Spain; 3 Department of Anatomy, Campbell University School of Osteopathic Medicine, Buies Creek, NC 27506 USA; 4 Evolutionary Studies Institute, University of the Witwatersrand, Private Bag 3, Johannesburg, WITS 2050 South Africa

**Keywords:** Biological anthropology, Palaeontology

## Abstract

Two well-preserved, subadult 800 ky scapulae from Gran Dolina belonging to *Homo antecessor*, provide a unique opportunity to investigate the ontogeny of shoulder morphology in Lower Pleistocene humans. We compared the *H. antecessor* scapulae with a sample of 98 *P. troglodytes* and 108 *H. sapiens* representatives covering seven growth stages, as well as with the DIK-1-1 (Dikika; *Australopithecus afarensis*), KNM-WT 15000 (Nariokotome; *H. ergaster*), and MH2 (Malapa; *A. sediba*) specimens. We quantified 15 landmarks on each scapula and performed geometric morphometric analyses. *H. sapiens* scapulae are mediolaterally broader with laterally oriented glenoid fossae relative to *Pan* and Dikika shoulder blades. Accordingly, *H. antecessor* scapulae shared more morphological affinities with modern humans, KNM-WT 15000, and even MH2. Both *H. antecessor* and modern *Homo* showed significantly more positive scapular growth trajectories than *Pan* (slopes: *P. troglodytes* = 0.0012; *H. sapiens* = 0.0018; *H. antecessor* = 0.0020). Similarities in ontogenetic trajectories between the *H. antecessor* and modern human data suggest that Lower Pleistocene hominin scapular development was already modern human-like. At the same time, several morphological features distinguish *H. antecessor* scapulae from modern humans along the entire trajectory. Future studies should include additional *Australopithecus* specimens for further comparative assessment of scapular growth trends.

## Introduction

The TD6.2 level of the Gran Dolina cave site (Sierra de Atapuerca, Burgos Spain) has provided about 170 human remains belonging to a minimum of eight individuals^1,2^. These fossils have been dated to the late Lower Pleistocene (between 772 and 949 ky) and exhibit a unique combination of cranial, mandibular, and dental features. To accommodate the variability observed in the TD6 human fossils a new species of the genus *Homo*, *H. antecessor*, was proposed in 1997^3^. These human remains are well preserved, although fragmented probably due to at least two cannibalism events^4–6^. The human remains, as well as more than 831 lithic instruments^7^ and several thousand fossil remains of different species of micro and macrovertebrates were obtained during two different periods, 1994–1996 and 2003–2007, in the excavation of two sections of the TD6 level^8,9^. The last geological study of the TD6.2 sequence has been made by Campaña et al.^10^. A recent study of the enamel proteins recovered from a molar fragment (ATD6-92) suggests that *H. antecessor* is a closely related sister taxon of the last common ancestor of *H. sapiens*, *H. neanderthalensi*s and Denisovans^11^.

Among all the fossils yielded by the stratum TD6, cranial and postcranial remains have been studied during the last decades^9,12–16^. *H. antecessor* is rather unique among fossil *Homo* taxa, in that its postcranial morphology shares several derived features found in both modern humans and Chinese Middle Pleistocene hominins^1^. The latest research published on the evolutionary anatomy of the skeletal anatomy of this species is a descriptive study of the immature scapulae ATD6-116 and ATD-118. The right ATD6-116 scapula was recovered in 2005 in the square G14, whereas the right ATD6-118 scapula was recovered in two different years (2006 and 2007) in the square F12^17^. This study showed that the *H. antecessor* scapular morphology had the general pattern of the genus *Homo*, with minor differences compared to *H. sapiens*^17^. However, the authors did not investigate whether scapular development in the Gran Dolina hominins was similar to *H. sapiens* or followed a more primitive trend.

### Evolutionary development in the context of the human scapulae

Adult shapes are the result of a multitude of developmental events beginning in utero and through the postnatal periods^18^. Even though most of the changes in the organisms are largely in place by birth^19^, postnatal growth and development (postnatal ontogeny) are crucial to understanding the adult form of the different skeletal regions since a huge number of size and shape changes occur in the human body during those phases^20–26^. Young^27,28^ investigated morphological changes in a large ontogenetic series of hominoid scapulae, suggesting that prenatal and early postnatal development are key to understanding adult scapular morphology since most of the distinctive interspecific features were already present in infants. Those features included the shape of the scapular blade and the glenoid fossa, the angle of the spine relative to the axial and vertebral border, and the length of the scapular spine, suggesting that postnatal ontogenetic development only accentuates the features already set prenatally or established during an early postnatal stage^28^.

From an evolutionary point of view, the study of developmental changes that occurred during prenatal and postnatal ontogeny could provide important information of phylogenetic interest^19,24,29,30^. Adult morphologies can vary between species because of (1) differences in the shape of the anatomical element at the moment of birth that is caused by differences in the prenatal ontogenetic trajectories; (2) differences in the shape of the anatomical element that arise after birth and are caused by differences in the postnatal ontogenetic trajectories; and (3) differences in the magnitude of the ontogenetic change after birth^25^. On the one hand, when the morphological differences between two closely related species are already found at birth but the postnatal morphological changes are the same in both, we can say that their ontogenetic trajectories are parallel^24,25,31^. Alternatively, when the two species are similar at birth but undergo different postnatal changes, their ontogenetic trajectories are divergent^24,25,31^. This distinction is important because if postnatal ontogenetic trajectories are parallel between both species it would suggest consistency of genetic regulation^25^. Furthermore, two evolutionary-related species (basal and terminal) having the same ontogenetic trajectory would greatly impact the determination of traits as being plesiomorphic or apomorphic for a given lineage^32,33^.

In his study of the ontogeny of the hominoid scapula, Young^28^ found that ontogenetic trajectories were similar across functional (e.g., knuckle-walkers) and phylogenetic groups (e.g. Atelidae) since features discriminating functional and phylogenetic groups were largely present in infants and changed little over the course of ontogeny. Even though it was not specifically addressed in his study, based on this observation, we hypothesize that scapular ontogenetic trajectories between similar functional and/or phylogenetic groups should be parallel, suggesting a consistency of genetic regulation^25^.

### Evolutionary anatomy of the human scapulae

Although comparative scapular ontogeny and morphology is relatively easy to evaluate among extant taxa^27,28,34^, extending these analyses to the fossil record is particularly challenging given the inherent bias in the preservation of this fragile skeletal element. The discovery of a complete set of scapulae of a ~ 2.5 year-old female *A. afarensis* individual from Dikika, Ethiopia (DIK-1-1; 3.4 Ma)^35–37^ promised to shed light on questions about postcranial development in this ancient hominin species. Green and Alemseged suggested that the DIK-1-1 scapulae were most similar to those of *Gorilla* juveniles^38^, and when considered alongside other adult australopith scapulae (e.g., KSD-VP-1/1 and A.L. 288-1), the growth of the *A. afarensis* shoulder may have followed an ape-like trajectory, which was supported by its dental development^36,39^. Analysis of another *A. afarensis* scapula from Woranso-Mille, Ethiopia (KSD-VP-1/1)^40^, a potential large male individual dated around 3.6 Ma^41^, did not present such an apelike model of the adult australopith shoulder blade^38,42–44^. The KSD-VP-1/1 scapula preserves more derived features relative to DIK-1-1, though differences between them do not appear to exceed the magnitude of ontogenetic variation that may exist within a living species (and across sexes)^42,43^. Specifically, the KSD-VP-1/1 scapula is different from that of African apes in having a less cranial orientation of the spine as well as features linked with a manipulatory function of the upper limb, such as the infraspinous fossa expansion. Further research on the Dikika specimen hypothesized its adult morphology using different ontogenetic primate trajectories and suggested an ape-like adult shape for it, in contrast to what was found in the KSD-VP-1/1 scapula^44^. In this context, “ape-like” refers to a mediolaterally narrow scapula with a cranially oriented glenoid and acromion, hypothesized as the plesiomorphic condition. At the very least, the *A. afarensis* shoulder was less derived than that of modern humans, conditions that may be even more pronounced in subadults relative to mature individuals.

The earliest representatives of the genus *Homo* comes from the complete subadult *H. erectus*/*ergaster* scapula from Nariokotome^45^ (Kenya, around 1.4 Ma^46^), fragmentary glenoids from Dmanisi^47^ (*H. georgicus*, ~ 1.8 Ma^48^), Flores^49^ (*H. floresiensis*, ~ 0.1 Ma^50^), and Rising Star^51^ (*H. naledi*, ~ 0.3 Ma^52^), and now, the nearly complete *H. antecessor* fossils from Gran Dolina^17^ (~ 0.8 Ma^53^). Although *H. naledi* and *floresiensis* are not “primitive” representatives in terms of chronology, both of these taxa demonstrate considerable “primitive” morphologies in addition to their taxonomic attribution to early *Homo*^54–56^.

The Dmanisi scapula (D4166) shows a glenoid cavity that is more cranially oriented than in modern humans and closer to *Australopithecus* and African great apes. Also, the narrow glenocoracoid angle and the high width-to-length ratio of the coracoid process clustered D4166 closer to great apes^47^. The only variable that associates it with a modern human morphology is the glenoid orientation relative to the spine and the breadth-to-width ratio of the spine, which is also similar to the Nariokotome specimen^47^. The authors concluded that the Dmanisi had more australopith-like than human-like scapular morphology^47^. The *H. naledi* scapula had a cranially-oriented glenoid cavity, similar to that of *Australopithecus* and distinct from that of modern humans^57^; Feuerriegel et al. reconstructed the clavicle as relatively short, potentially indicating that the scapula was located more superiorly and laterally about the thorax.

In contrast, the *H. erectus*/*ergaster* scapula from Nariokotome represents the earliest clear evidence of derived scapular morphology, similar to that of modern humans^38,45,49^. The anatomically derived features include a more transverse scapular spine orientation and a more lateral-facing glenoid cavity with respect to that seen in *A. afarensis*^38,49^*.* Most recently, the scapulae from Gran Dolina also present derived, modern humanlike morphology compared to *Australopithecus*. These features include relative supra- and infraspinous breadths, infrascapular breadth/glenoid size, scapular spine orientation, and glenoid orientation^17^. Importantly, some similarities were noted between Gran Dolina and Nariokotome that differentiate them from modern humans, such as the axillospinal angle and scapular index, leading to the possibility that Lower Pleistocene hominin scapulae could have been relatively narrower than those of modern humans.

These previously discovered scapulae provide important information about recent scapular evolutionary anatomy, but they do little to illuminate evolutionary development. While the Nariokotome specimen is subadult, modern scapulae from its stage of development (2nd molars erupted) differ modestly in size and shape from those of mature individuals. Moreover, the scapular material from other early *Homo* fossil sites covers a wide chronological and geographical range, and their association to the same group (early *Homo*) is far from certain^58^. Alternatively, the Gran Dolina material provides a unique opportunity to study evolutionary scapular growth and development from the same Lower Pleistocene *Homo* species – essentially two individuals from the same population, from a geological perspective.

### Aims of this work

In this study, we seek to evaluate the antiquity of modern-like scapular development. Previously, such a question has been difficult to investigate, as both primitive^47,57^ and derived^17,38,45,49^ morphologies have been proposed for the scapulae of early *Homo* fossil sites. Furthermore, the limited fossil record has precluded any consideration of ontogeny in general or in a comparative context. In the light of the recent discovery of fairly well-preserved subadult *H. antecessor* scapulae, we aim to test the hypothesis that Lower Pleistocene members of the genus *Homo* followed a modern-like pattern of scapular development.

## Results

### Size and growth of *H. antecessor* scapulae

**Figure 1:**
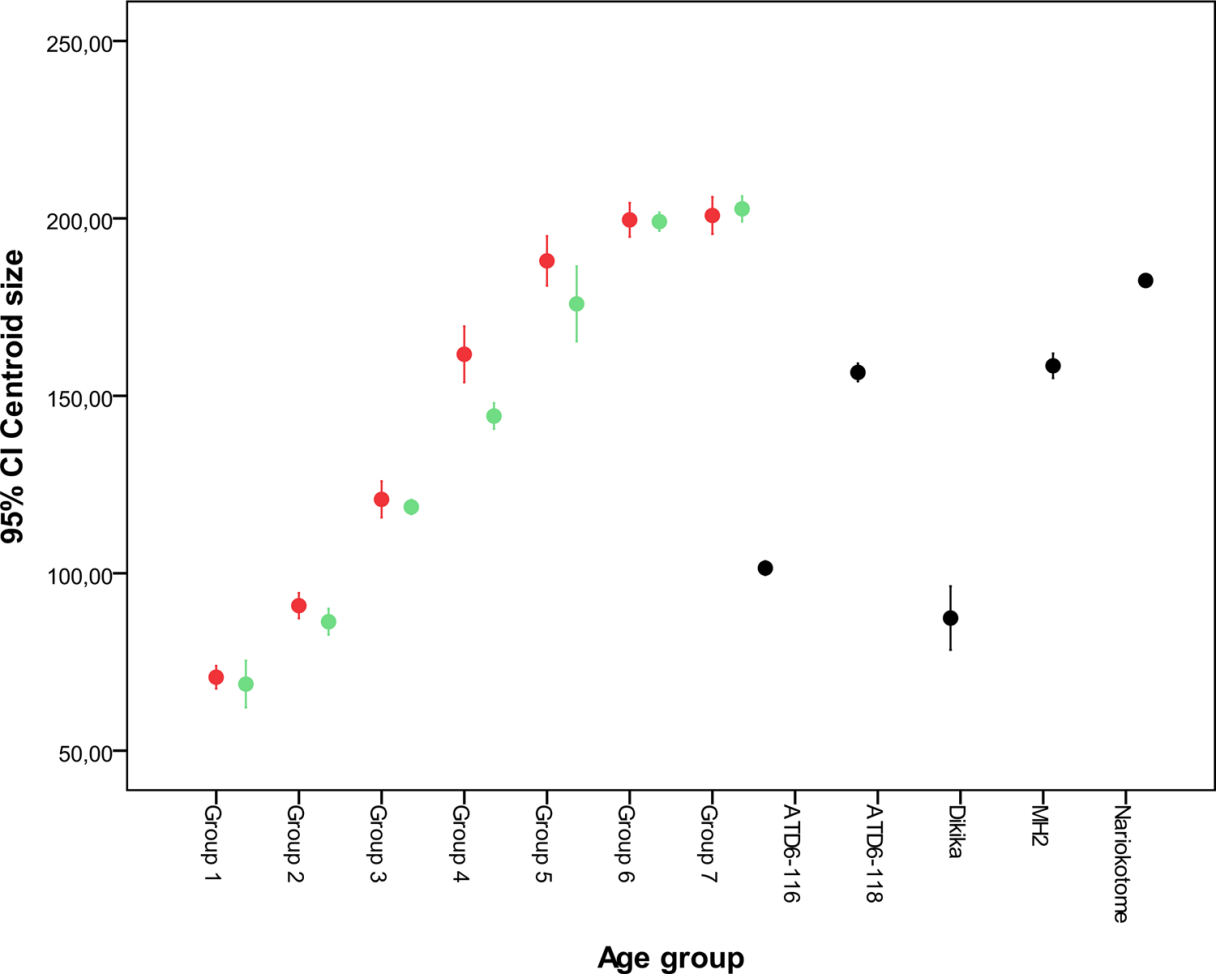
Centroid size analysis for the current and fossil specimens, showing 95% confidence intervals for the mean of each species and group. Numbers along the x-axis refer to the ontogenetic stage in *Pan* and *Homo* (green = *Pan*; red = *Homo*) and fossil scapulae are inserted. The DIK-1-1 interval is based on its right and left scapula; other fossil CI are based on particular estimates from previous analyses. DIK-1-1 is stage 2, based on its fully erupted deciduous dentition (no permanent teeth). KNM-WT 15000 is between stages 4 and 5, based on its fully erupted 2nd molar. MH2 is a small adult female, based on long bones lacking an unfused epiphysis^65^. The Gran Dolina fossils are not associated with dental remains, and so their ontogenetic stage can only be estimated by their relative size.

The ATD6-116 scapula falls between the centroid size of groups 2 and 3 from both modern humans and chimpanzees, being slightly larger than the DIK-1-1 scapulae (Fig. 1). The ATD6-118 specimen falls within the size of group 4 from modern humans, very close to the size of the MH2 scapula, and being smaller than the Nariokotome scapula (KNM-WT 15000). The growth (size increase with age) shows that humans and chimpanzees have a similar growth pattern in stages 1–3 and 6–7, but humans have a more rapid growth in stages 3–4, which slows between stages 5–6.

### Morphology and development in *H. antecessor* scapulae

**Figure 2:**
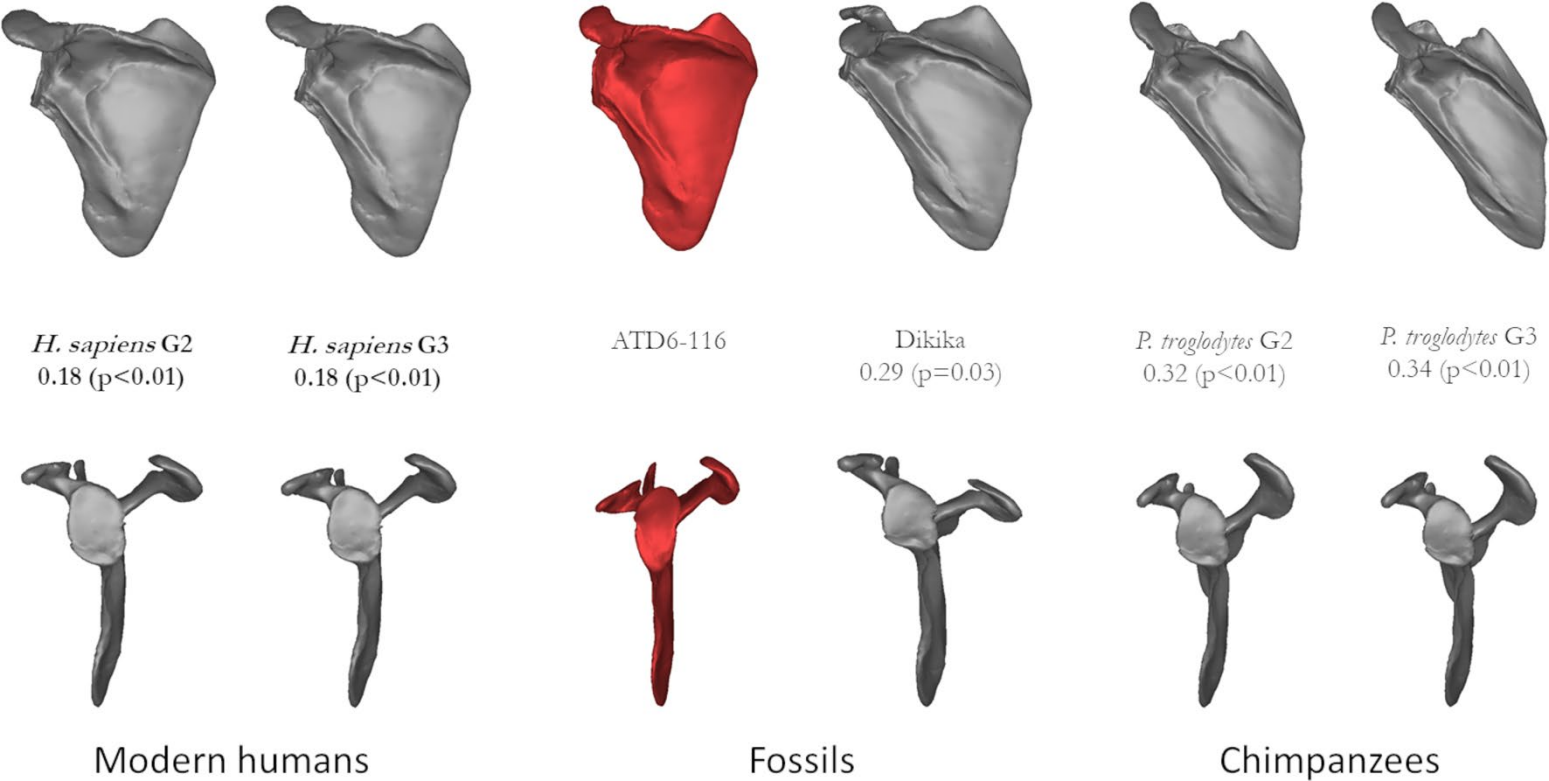
Mean shape comparisons from ATD6-116 (center, red) next to the most similar groups based on size and (probable) developmental stage. The Procrustes distance from ATD6 to each group is shown as well as the result of the permutation test. Note that the distal-most point of the acromion is hypothetically depicted here as it is not fully ossified in individuals of this age stage. The distal-most ossified point was the captured landmark across the comparative sample (see also Table S1).

**Figure 3:**
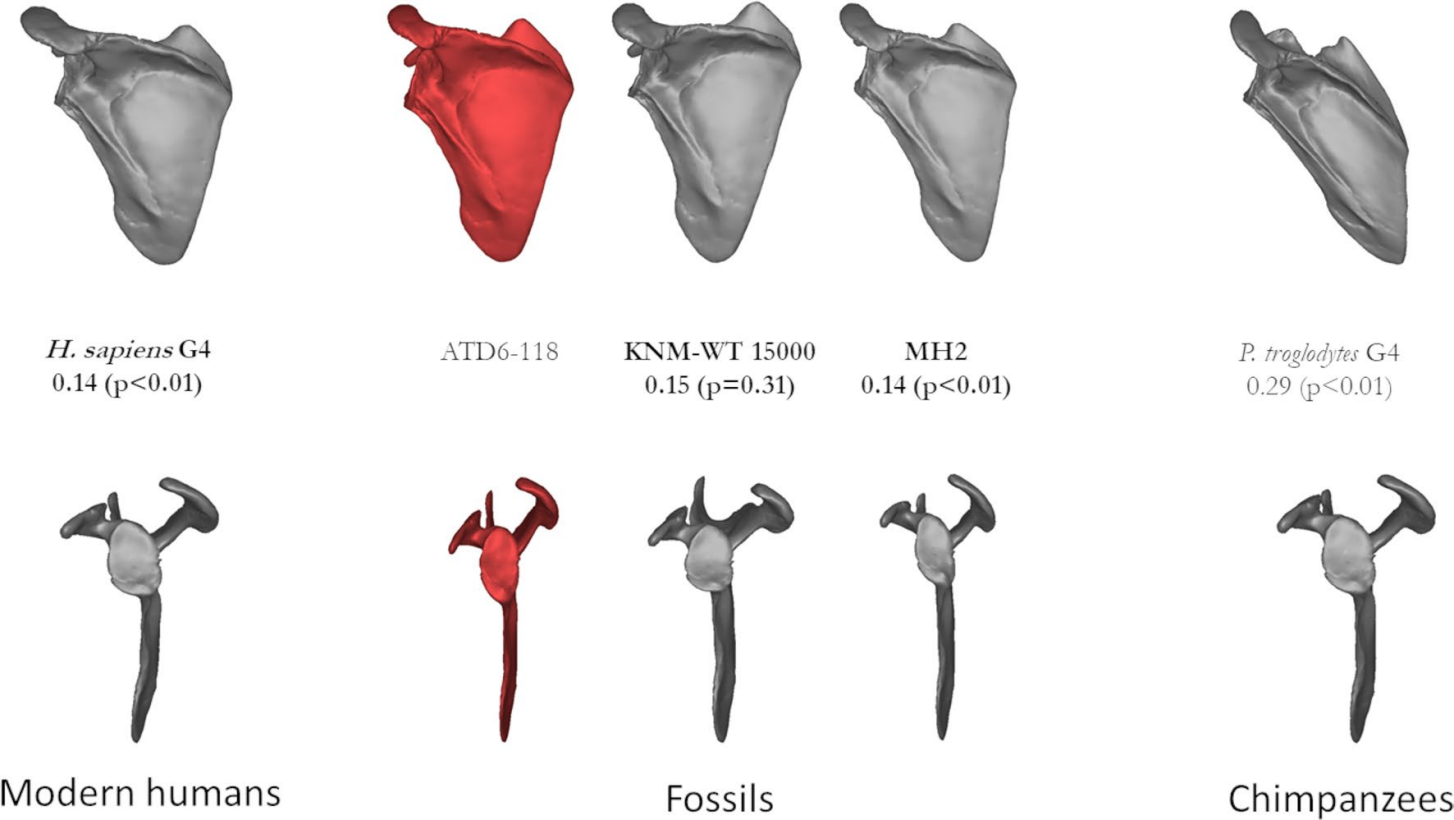
Mean shape comparisons from ATD6-118 (left center, red) next to the most similar groups based on size and (probable) developmental stage. The Procrustes distance from ATD6 to each group is shown as well as the result of the permutation test.

When we compared the shape of *H. antecessor* scapulae with individuals from similar age stages in terms of size, we found that the shape of the ATD6-116 specimen is more similar to stage 2 and 3 from *H. sapiens* than to the Dikika specimen, and stages 2 and 3 from *P. troglodytes* (Fig. 2). The relative width/height ratio accounts for most of these similarities, but the glenoid, spine, and acromion orientation also aligns the ATD6-116 with humans. The shape of ATD6-118 is more similar to all hominins (modern human groups 4 and 5, KNM-WT 15000, MH2) than to chimpanzees, *H. sapiens* group 4, and MH2 being the closest groups to the shape of ATD6-118 (Fig. 3). The relative width/height ratio accounts for most of these differences, as well as the glenoid fossa orientation. In contrast to what we found for the younger specimens, the acromion and spine orientation did not differ dramatically across these specimens, but it is important to state that this part unfused (and thus estimated) in most of the youngest individuals, including ATD6-116. Therefore, the interpretation of this particular feature in very young specimens should be taken with some caution.

**Figure 4:**
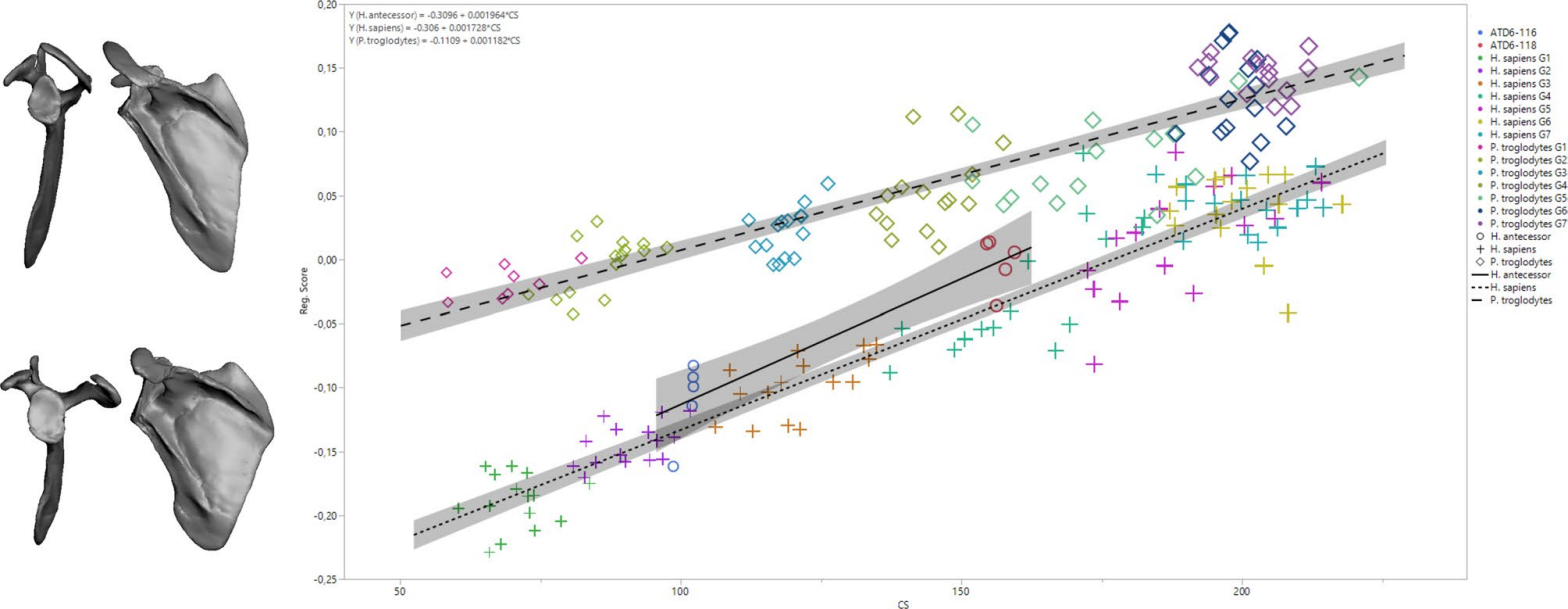
Regression analysis of full shape on size showing ontogenetic trajectories for *H. sapiens* (“+” symbols), *P. troglodytes* (“diamond” symbols), and *H. antecessor* (“circle” symbols). The regression formula and the confidence intervals are shown. Notice that for *H. antecessor,* all the acromion estimations are depicted (see “Materials and methods”). Different colors depict different age groups.

**Figure 5:**
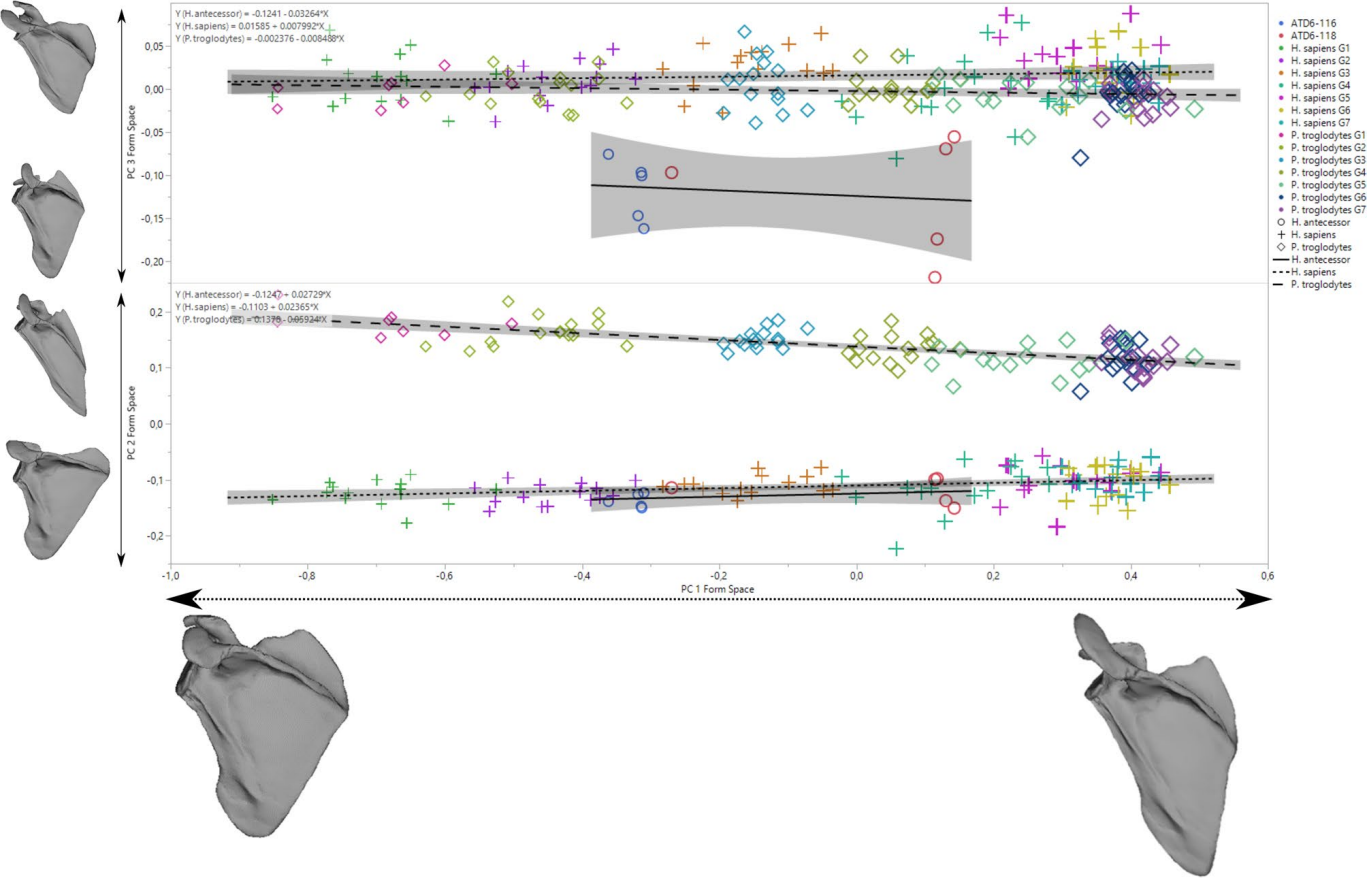
Form space PCA showing ontogenetic trajectories for *H. sapiens* (“+” symbols), *P. troglodytes* (“diamond” symbols), and *H. antecessor* (“circle” symbols). The regression formula and the confidence intervals are shown. Notice that for *H. antecessor,* all the acromion estimations are depicted (see “Materials and methods”). Different colors depict different age groups.

**Figure 6:**
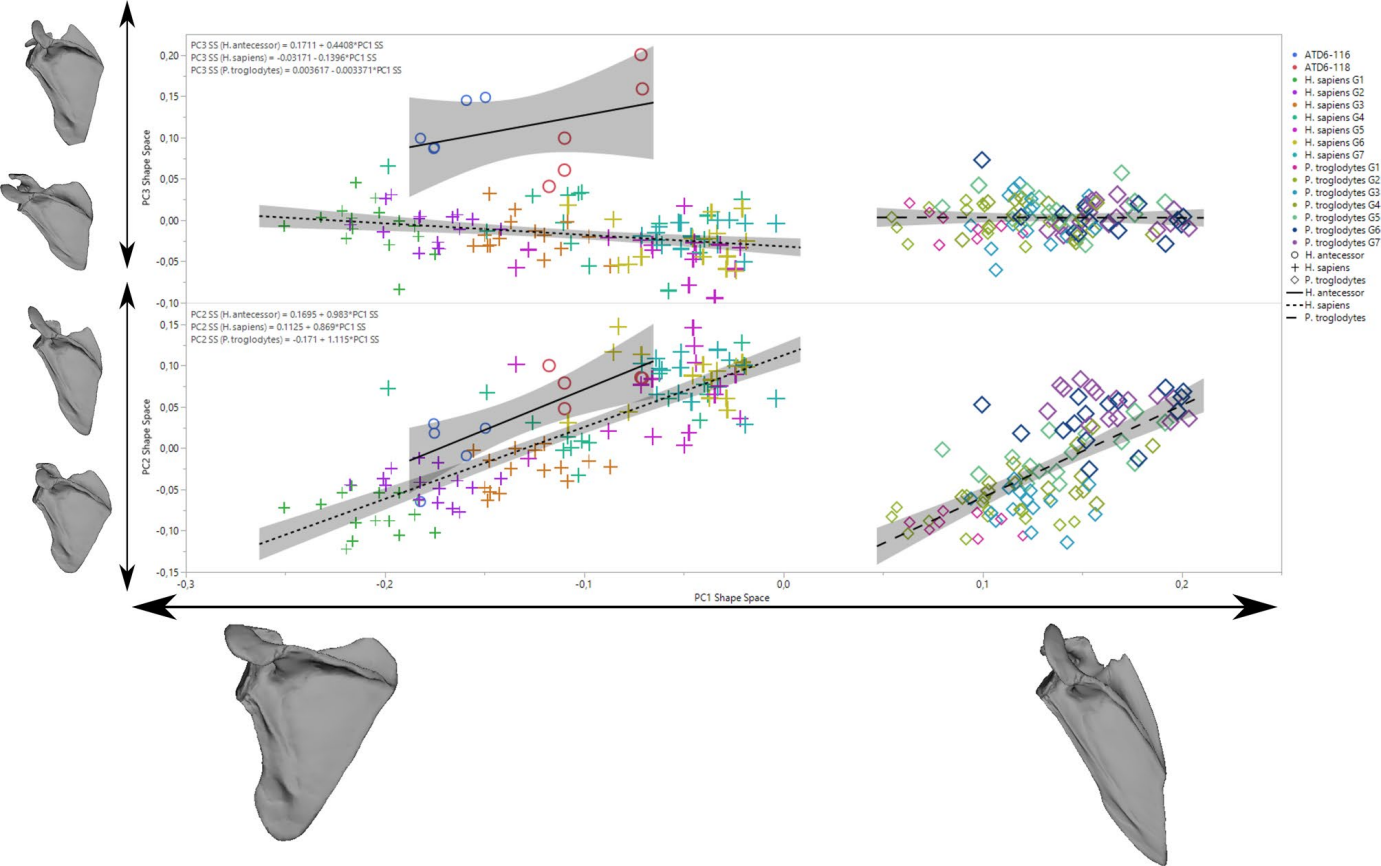
Shape space PCA showing ontogenetic trajectories for *H. sapiens* (“+” symbols), *P. troglodytes* (“diamond” symbols), and *H. antecessor* (“circle” symbols). The regression formula and the confidence intervals are shown. Notice that for *H. antecessor,* all the acromion estimations are depicted (see “Materials and methods”). Different colors depict different age groups.

In the regression analysis of shape on size (here a loose proxy for age) to assess developmental changes, we observe a slight increase in relative height with age (Fig. 4). However, it is important to note that the rate of change appears to be faster in modern humans and fossil hominins relative to chimpanzees, based on the higher slope of the regression of shape on the size of modern humans (slope: 0.0018; 95% CI 0.0017–0.0019) and *H. antecessor* (0.0020) compared to chimpanzees (slope: 0.0012; 95% CI 0.0011–0.0013). When ontogeny is decomposed through the study of the individual components in a form space PCA (Fig. 5; Fig. S2), we observe similar trends in PC1 and PC2, but PC3 shows that *H. antecessor* is polarized in the negative values of this score (PC3 scores—human 95% CI 0.009, 0.021; *Pan* 95% CI − 0.007, 0.002; ATD6-116 mean − 0.12; ATD6-118 mean − 0.12; Fig. 5). This uniqueness can be also observed in the PC3 of shape space (> 5% of the variability of the sample), where *H. antecessor* again plots far from the distribution of humans and *Pan* (Fig. 6; Fig. S3). This would suggest some unique features in the fossil hominin ontogenetic trend that are observed neither in modern humans nor in apes, which are likely plesiomorphic with respect to Lower Pleistocene *Homo*. Some of these features include the superior-inferior relative length of the blade as well as the position of the superior angle. Some features can be also related to the acromion, but since this structure is estimated in the youngest specimens, we should be cautious interpreting that part of the scapula.

### Ontogenetic trajectories of current species and *H. antecessor*

The ontogenetic trajectories, assessed as full shape changes on size, show that the modern human and chimpanzee trends are convergent (Fig. 4). This means that there are larger differences in early ontogenetic stages than in later ontogenetic stages. Even though the ontogenetic trajectory of *H. antecessor* has more positive regression score values than the modern human trajectory (Figs. 4, 5, 6), its slope is much closer to that of modern humans than chimpanzees (Figs. 4, 5, 6).

## Discussion

*Homo antecessor* is a Lower Pleistocene *Homo* species from the Iberian Peninsula dated around 0.8 Ma. Its skeletal morphology is key for understanding human evolution; given its proximity to the separation of the Neanderthal and modern human lineage, *H. antecessor* is a reasonable candidate for the last common ancestor of *H. sapiens*, *H. neanderthalensi*s and Denisovans^11^. The large skeletal assemblage found in level TD6.2 provides a unique opportunity to study the evolutionary anatomy of different parts of the human body in an early human species. Its scapular anatomy has been recently described as closer to humans than to great apes^17^, and here we tested the hypothesis that the development of *H. antecessor* was similarly human-like, by comparing its ontogenetic trajectory with that of *P. troglodytes*, *H. sapiens*, and the genus *Australopithecus*, as represented by individuals from *A. afarensis*^37^ and *A. sediba*^59^.

### Scapular size and growth in *H. antecessor* compared to current and fossil species

Based on the epiphyseal fusion^60^, it was estimated that if ATD6-118 belonged to a modern population, it would be younger than approximately 14–15 years old^17^. However, alternative estimates based on maximum scapular length yielded an age of approximately between 16 and 22 years^61^. Based on those same measurements, they hypothesized that ATD6-116 could belong to a 2–4 years old individual. However, as we see in our results, all the fossil hominin scapulae, except that of Dikika, are relatively taller compared to that of *H. sapiens*, so the maximum scapular length could drive to misleading conclusions in fossils. In this study, we compared the centroid size of the *H. antecessor* scapulae with ontogenetic samples of *H. sapiens* and *P. troglodytes*, from developmental groups categorized by dental eruption sequence. The ATD6-116 scapula is slightly larger than the Dikika specimen and falls between dental stages 2 and 3 from both *H. sapiens* and *P. troglodytes*. To that end, we feel comfortable presuming that this individual may have at least had all deciduous teeth fully erupted or additionally presented a partial or fully erupted permanent M1. Applying these criteria to the ATD6-118, we observe that it is very close to group 4 of both humans and chimpanzees whereas Nariokotome boy (dental stage 4–5) is closer to group 5, based on centroid size. Therefore, we assume that the ATD6-118 individual had the M2 partially or fully erupted. The fact that the Nariokotome scapula falls closer to group 5, defined by “erupting canine and/or M3”, is consistent with research about his dental development. This is because this individual had 26 permanent teeth emerged, all but third molars and upper canines and wear on teeth suggested that the lower canines were probably the last teeth to erupt before death^62^.

### Scapular shape and development in *H. antecessor* compared to current and fossil species

Comparing the shape of the *H. antecessor* scapulae with like-aged specimens (based on size) demonstrates that the shape of the ATD6-116 specimen is more similar to *H. sapiens* than to the Dikika specimen and chimpanzees (Fig. 2). The orientation of the glenoid fossa in Dikika is more cranial than in *H. antecessor* and is similar to that of chimpanzees, which is consistent with previous research^37,38,42,63^, but it is also important to state that the Dikika scapulae have some important differences compared to that of chimpanzees. Specifically, the overall shape quantified by the height/width ratio is much more similar among DIK-1-1, ATD6-116, and modern humans relative to subadult chimpanzees. The shape of the larger ATD6-118 scapula is also closer to modern humans and the other fossil specimens, Nariokotome and MH2, than chimpanzees (Fig. 3). As before, the relative height/width ratio accounts for most of these differences. It is interesting to note that the *Australopithecus sediba* scapula is more similar to Lower Pleistocene *Homo* than the *A. afarensis* scapula of Dikika, based on Procrustes distance. In contrast, the Procrustes distance between Dikika and ATD6-116 is greater (0.29) than that of MH2 and ATD6-118 (0.14) (Figs. 2, 3). Both current and fossil hominins are more similar to one another and different from the chimpanzee scapula in terms of height/width ratio. Yet still, it is important to note some differences between *H. sapiens*, *H. erectus/ergaster,* and *H. antecessor*, compared with *A. sediba*. This includes the more cranial orientation of the glenoid fossa and acromion in *A. sediba* compared to the *Homo* group, which could be linked to the archaic morphology proposed for the *A. sediba* upper limb and thorax^64,65^. Since previous researchers indicated that the shape of the adult scapula was tightly associated with positional behavior and locomotion^27,28^, the differences observed here can be linked to locomotor behavioral differences between *Australopithecus* and *Homo*. To this end, the lack of obvious arboreal adaptations in the Gran Dolina specimens suggests a likely abandonment of arboreal activities in *H. antecessor*, in contrast to the pattern observed in *Australopithecus.*

Regarding scapular development (changes in shape with age), previous research suggested that postnatal ontogenetic development only accentuates the features already present prenatally or at an early postnatal stage^28,30,66^. However, it is interesting to note here that the scapular development of chimpanzees and humans is convergent, in that juvenile human and chimpanzee morphology is more divergent relative to that seen in older individuals. This could be related to a more arboreal behavior in young chimpanzees than in adults, as observed by other researchers^67^. We also found that the developmental changes in hominins are largely comparable with one another; despite morphological differences, their regression slopes are very similar to each other (Fig. 4), implying similar developmental trajectories. In contrast, the hominin pattern was notably different from that of apes. This hypothesized pattern of scapular growth in *H. antecessor* is consistent with previous research on dental development^68^. At the same time, slight divergences observed in the regression analysis may be reflected in the differences observed in the PC2/PC3 axes from form and shape space analysis (Figs. 5, 6), which showed unique features of Lower Pleistocene *Homo*. These features include a superior projection of the scapular blade, combined with a slightly cranially oriented glenoid fossa and acromion.

### Limitations and future horizons

Even though dental methods are arguably the most efficient or practical approach for classifying ontogeny, we acknowledge that some assessments are subjective, especially when applied to samples combining distinct hominin species. This issue could be partially solved using documented populations, where exact ages-at-death are known in populations, and this should be used in future studies. Besides, we recognize that the *Australopithecus* record is very scarce, and we only evaluated morphological differences with the rest of the groups, but we did not attempt to study ontogenetic trends in *Australopithecus*. Future studies combining the information presented here with that of the MH1 juvenile (*A. sediba*^65^) and the KSD-VP-1/1 adult (*A. afarensis*^63^) would help to shed additional light on ontogenetic trends in this genus, and those two species, in particular. These two specimens were difficult to access, and the *H. naledi* scapula is not sufficiently well preserved to be included in our analyses. It would also be informative to include later fossil hominins from the Neanderthal lineage to explore the scapular development of this group in the light of the ontogenetic basis established here. Furthermore, even though our landmarks approach (15 landmarks) cover accurately the gross scapular morphology, future studies could improve the landmarks quantification through the use of semilandmarks, a quantification technique extensively increasing in the field of paleoanthropology^69–73^.

Finally, our ontogenetic analysis addresses evolutionary morphology but we do not directly investigate functional morphology in fossil species, since this would require the use of specific comparative functional (e.g., locomotion) data. The incorporation of analyses of functional morphology could evaluate/test the functional significance of our findings following 3 potential avenues for future research: (a) Biomechanical 3D models applied on early hominin fossils^74,75^, (b) trabecular morphology, widely used in functional morphology studies^76–78^, and (c) refined analysis of 3D muscle attachment sites (entheses)^79^, considering supportive experimental evidence involving laboratory animals^80,81^. Our work opens up a new window for addressing all these issues in fossil hominins from the Gran Dolina site in future studies.

### Conclusions on evolutionary development in Lower Pleistocene Homo and Australopithecus

Our results suggest that, despite slight differences, the scapular morphology of *H. antecessor* was much more similar to modern humans than to great apes. In addition, the ontogenetic trajectory of *H. antecessor* and *H. sapiens* were nearly parallel, suggesting that the growth and development of *H. antecessor* were more similar to that of *H. sapiens* than to great apes. Even though we are conscious of the limitations of the sample size provided by the fossil record of Lower Pleistocene hominins, our data suggest that the trend towards a modern human-like scapular development would have occurred at least 0.89 Ma ago as demonstrated by *H. antecessor*.

## Materials and methods

### Sample composition and data acquisition

Background information regarding the ATD6, KNM-WT 15000, *A. afarensis,* and *A. sediba* scapulae can be found in the corresponding literature^17,37,45,65^*.* Data acquisition of original scapular material from the ATD6 fossils to produce 3D models was carried out through a V|Tome|X s 240 (GE Sensing & Inspections Technologies) under a resolution of 90 microns at the Centro Nacional de Investigación sobre la Evolución Humana (CENIEH) facilities. The 3D model of the MH2 *A. sediba* scapula was downloaded from the Morphosource website (https://www.morphosource.org/; Identifier: S3451). Landmarks for the Dikika and Nariokotome specimens were digitized on the original fossils housed at the Ethiopian National Museum and National Museums of Kenya, respectively using an Immersion MicroScribe G2 digitizer. MicroScribe and digital methods have been successfully combined in other studies, finding no significant error between the two of them^82^.

The comparative human and chimpanzee samples comprise 105 and 98 scapulae respectively, from different age groups on specimens housed at the National Museum of Natural History (Washington, DC), the American Museum of Natural History (New York City), the Cleveland Museum of Natural History (Ohio), the Museum of Comparative Zoology (Cambridge, MA), and the Powell Cotton Museum (Birchington, UK). Individuals were sorted into seven age categories based on dental eruption and cranial suture fusion as follows: 1—deciduous teeth not fully erupted; 2—all deciduous teeth fully erupted; 3—deciduous dentition and partial or fully erupted M1; 4—M2 partially or fully erupted; 5—erupting canine and/or M3; 6—young adult, full permanent dentition, basioccipital suture open, little tooth wear; 7—full adult, full permanent dentition with basioccipital suture closed, moderate to heavy tooth wear^34,83^. A balanced sample of 15 individuals from each group both from *H. sapiens* and *P. troglodytes* was included, except for group 1 of *P. troglodytes*, which included 8 individuals. Landmarks were digitized for these specimens using an Immersion MicroScribe G2 digitizer, as was carried out for the Dikika and Nariokotome fossils. Further details of the sample are listed in Table 1.

**Table 1:** Extant comparative sample by age and sex (U = unknown, M = male, F = female, Total).

	1	2	3	4	5	6	7	Total
*Pan troglodytes*	(2U, 3 M, 3F, **8**)	(1, 8, 6, **15**)	(5, 6, 4, **15**)	(4, 6, 5, **15**)	(0, 9, 6, **15**)	(0, 8, 7, **15**)	(0, 9, 6, **15**)	98
*Homo sapiens*	(8U, 6 M, 1F, **15**)	(12, 1, 2, **15**)	(6, 4, 5, **15**)	(2, 4, 9, **15**)	(1, 7, 7, **15**)	(0, 8, 7, **15**)	(0, 9, 6, **15**)	105

Comparative human data was derived from Native American collections housed at the American Museum of Natural History (AMNH; New York, NY) and the National Museum of Natural History (NMNH; Washington, DC) and industrialized populations from the Cleveland Museum of Natural History (CMNH; OH) and NMNH. Chimpanzee data was collected from the Museum of Comparative Zoology (Cambridge, MA), the Powell-Cotton Museum (Birchington, UK), AMNH, CMNH, and NMNH. Further information about age stage criteria may be found in the main text, Shea (1986) and Green (2013).

### Landmarks digitalization protocol

**Figure 7:**
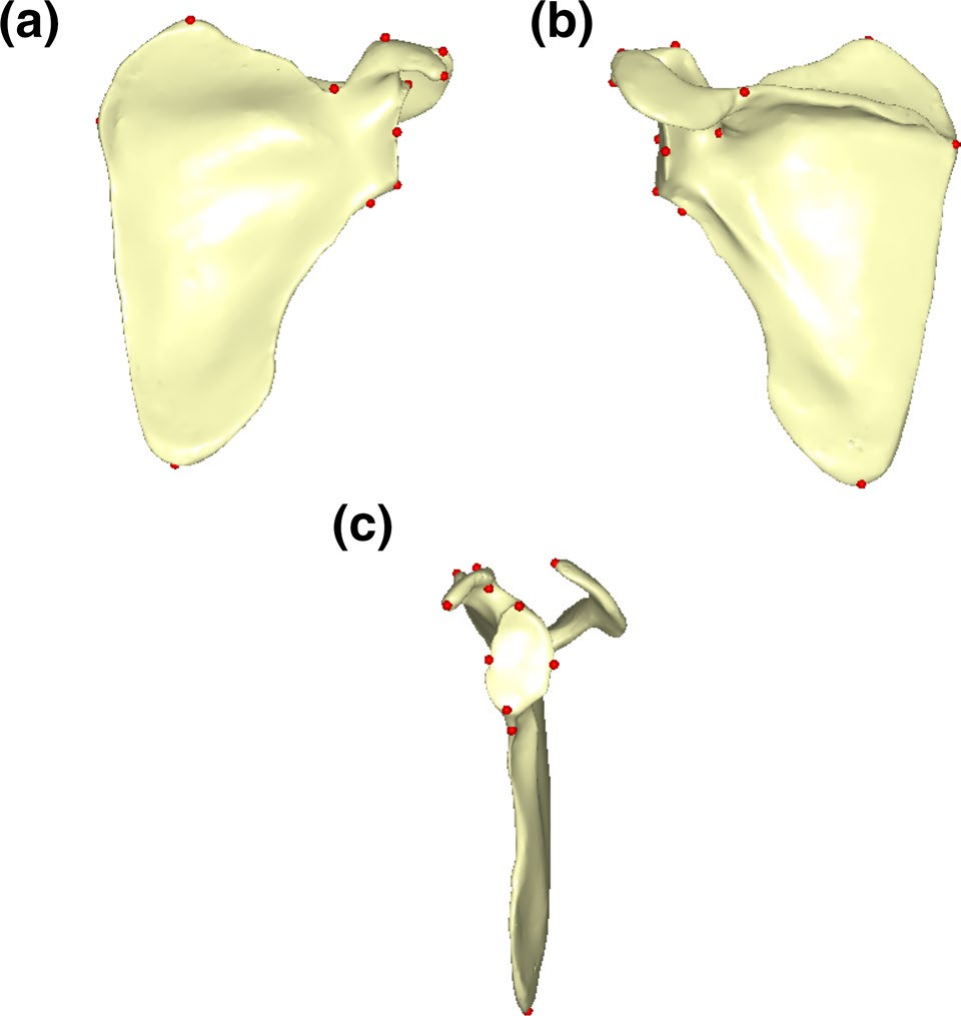
Landmarks configuration used in the study. Different views are observed, (**a**) internal view, (**b**) external view, (**c**) frontal view.

The scapulae from ATD6 were segmented through a semi-automatic protocol for DICOM images using the 3D Slicer software (https://www.slicer.org/) and subsequently reconstructed as 3D models. These 3D models were imported into Viewbox4 software (www.dhal.com)^84^ for landmarking using existing protocols^34^. The rest of the sample was digitized with an Immersion MicroScribe G2 digitizer. Scapular morphology was quantified through 15 homologous 3D landmarks measured in the blade, the glenoid fossa, the spine, and the acromion (Supplementary Online Material Table S2, Fig. 7 from the main text). In some fossils and subadult individuals, the coracoid process and acromion points were missing, so we carried out a TPS-based missing data estimation protocol using the most-similar/parsimonious individual as a reference^85^. Specifically, for the current samples, we were able to use as a reference the average coordinates of the complete individuals from each group and species for groups 2–7 (e.g., missing acromial points of individuals from *H. sapiens* group 2 were calculated using as a reference the average of the *H. sapiens* group 2 individuals that preserved that points). For group 1, since none of the individuals had those points preserved, we used the average of their respective groups 2 as a reference. For the fossil specimens, we followed a multi-approach that included the estimation of missing data using a wide range of references that included the most similar *H. sapiens* and *P. troglodytes* groups in terms of preserved landmarks as well as the most similar fossil specimen in terms of size that preserved those landmarks. For example, the ADT6-116 scapula landmarks were estimated using (1) the average of the *Pan* groups 2 and 3; (2) the average of the *Homo* groups 2 and 3; (3) the Dikika specimen as a reference (SOM, Figure S1). All of the estimated landmark configurations were analyzed to encompass the wide range of possible morphological reconstructions, as required by classic missing data estimation protocols^85^.

Intraobserver error testing of landmark placement had been carried out on this sample and reported in a previous study: “one adult male chimpanzee specimen was measured seven additional times over the course of several weeks. The average percent error was 1.6%, while individual average measurements ranged from 5.1% less than and 2.3% greater than the original measurement that was used in the subsequent analyses”^34^.

Once the whole set of coordinates were obtained, the landmarks were submitted to a Procrustes superimposition (GPA) and were analyzed following standard procedures for size and shape analysis^86^ using EVAN Toolbox (version 1.71; http://www.evan-society.org/) and MorphoJ^87^. The shape was addressed using GPA coordinates, and the size was studied through the centroid size (CS; calculated as the square root of the sum of squared distances of all the landmarks from their centroid^88^). We carried out shape comparisons between the ATD6 fossils and the mean of the most similar groups to them in terms of size (as an approach for development) through Procrustes distance comparisons and a permutation test (permutations = 1000) in MorphoJ software. The ontogenetic changes were addressed through a Form space PCA, in which the size is studied as part of the variation, and regression analysis of full shape on size, as done in other ontogenetic studies^20,22–24,73,89,90^.

## Supplementary Information

Supplementary Information.

## Data Availability

All data needed to evaluate the conclusions in the paper are present in the paper and the Supplementary Materials. Additional data is available upon reasonable request to the authors.
