# Supplementary material for: Evolutionary development of the Homo antecessor scapulae (Gran Dolina site, Atapuerca) suggests a modern-like development for Lower Pleistocene Homo
Source: Sci Rep. 2021 Feb 18;11:4102. doi: 10.1038/s41598-021-83039-w (PMC7892855; doi:10.1038/s41598-021-83039-w)
Supplement: Supplementary file 1 — Supplementary Information. [file 41598_2021_83039_MOESM1_ESM.docx]

**Supplementary Online Material**

**Title:** Evolutionary development of the *Homo antecessor* scapulae (Gran Dolina site, Atapuerca) suggests a modern-like development for Lower Pleistocene *Homo*

**Authors:** Daniel García-Martínez^1,2*#^, David Green^3#^, José María Bermúdez de Castro^1#^

^1^ Centro Nacional para el Estudio de la Evolución Humana (CENIEH), Paseo Sierra de Atapuerca 3, 09002 (Burgos, Spain)

^2^ Departamento de Paleobiología, Museo Nacional de Ciencias Naturales (CSIC), José Gutiérrez Abascal 2, 28006 (Madrid, Spain)

^3^ Department of Anatomy, Campbell University School of Osteopathic Medicine, Buies Creek, NC 27506, USA.

^#^All Authors contributed equally to the manuscript.

**Figure S1.** Example of missing data estimation range shown in the case of ATD6-116. The black dots are the landmarks that were measured in the specimen whereas the blue, green and red were estimated using the most similar fossil, Pan and Homo sapiens average specimens. G2 and G3 means Group 2 and 3, respectively.


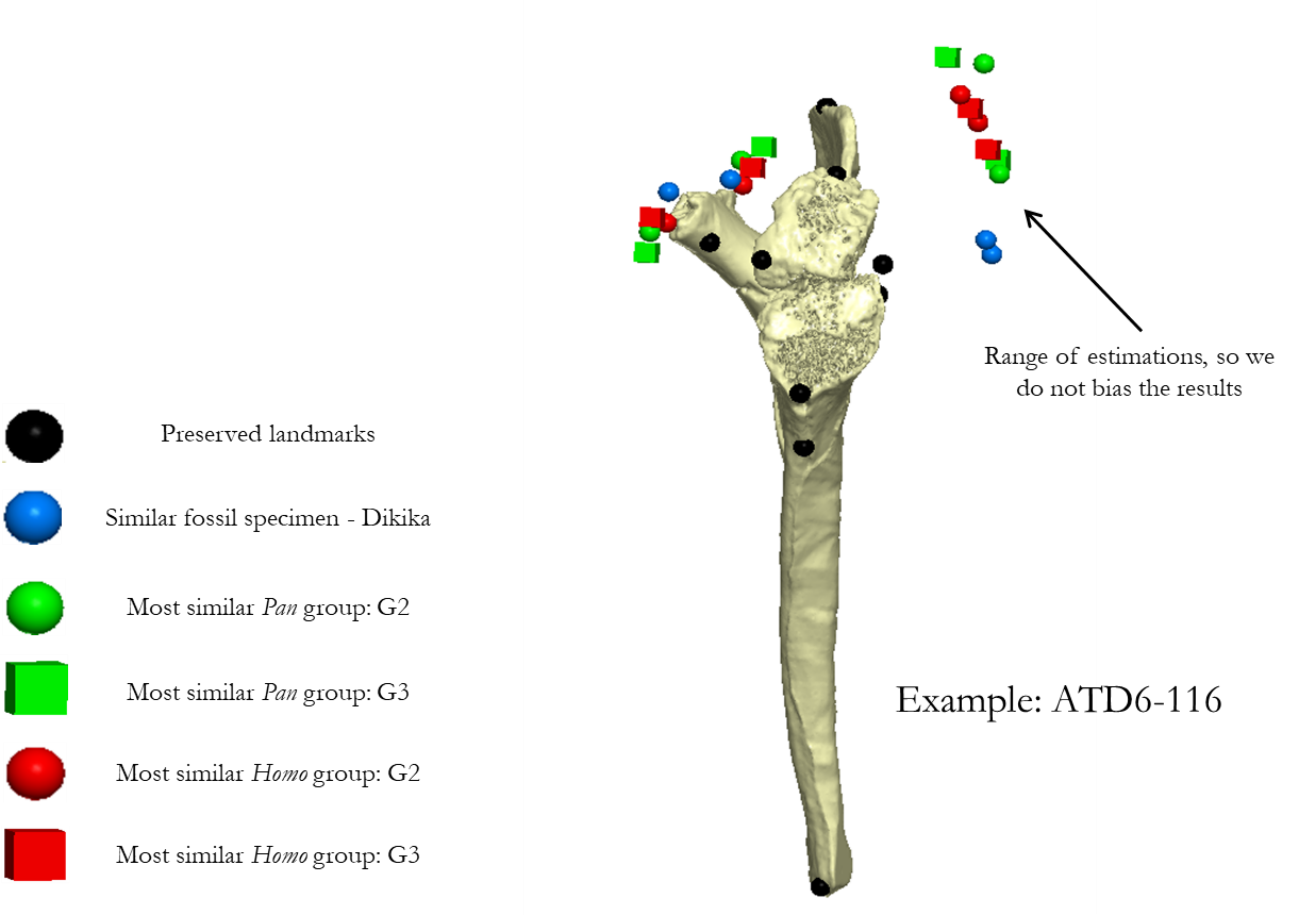


**Figure S2.** Scree plot of the Form Space PCA. Notice that PC3 is in the “elbow” of the curve.

**Figure S3.** Scree plot of the Shape Space PCA. Notice that PC3 is before the “elbow” of the curve.

**Table S1.** Scapular landmarks measured following the protocol from Green (2013).

| 1 | Suprascapular notch—the inferior-most point of this notch. |
| --- | --- |
| 2 | Superior angle—the superior-most point of the scapula with the superior and inferior angles in line with imaginary vertebral column. |
| 3 | Point of spine that meets vertebral border (a straight line from the spinoglenoid notch to the medial edge of the spine was this point if the spine diminished before intersecting with the vertebral border). |
| 4 | Inferior angle—the inferior-most point of the scapula with the superior and inferior angles in line with imaginary vertebral column. |
| 5 | Infraglenoid tubercle—inferomedial to point 7 (below); attachment site of triceps brachii muscle. |
| 6 | Spinoglenoid notch—the point where the base of the spine meets the blade inferior to the junction of the spine and the acromion. |
| 7 | Inferior-most point of glenoid fossa. |
| 8 | Coracoid side of maximum glenoid width—anterior-most point of fossa. |
| 9 | Acromion side of maximum glenoid width—posterior-most point of fossa. |
| 10 | Superior-most point of glenoid fossa. |
| 11 | Inferior “elbow” of coracoid—point directly below point 12 (below). |
| 12 | Superior “elbow” of coracoid—superior point where coracoid turns laterally. |
| 13 | Most distal point of coracoid. |
| 14 | Point of spine above spinoglenoid notch—the union of the spine and the acromion. |
| 15 | Distal most point on the acromion (distal-most ossified point in immature specimens). |
